# Attractiveness Ratings for Musicians and Non-musicians: An Evolutionary-Psychology Perspective

**DOI:** 10.3389/fpsyg.2019.02627

**Published:** 2019-11-29

**Authors:** Stephan Bongard, Ilka Schulz, Karin U. Studenroth, Emily Frankenberg

**Affiliations:** ^1^Department of Psychology, Goethe University Frankfurt, Frankfurt am Main, Germany; ^2^Division of Pediatric Neurology, Goethe University Frankfurt, Frankfurt am Main, Germany

**Keywords:** music, attractiveness, evolution, musician, courting behavior

## Abstract

From an evolutionary perspective, musical behavior such as playing an instrument can be considered as part of an individual’s courting behavior. Playing a musical instrument or singing might fulfill a function similar to that of a bird’s colored feathers: attracting attention. Therefore, musicians may be rated as more attractive than non-musicians. In an online survey, 137 volunteers (95 female) with ages ranging from 16 to 39 years rated the attractiveness of fictitious persons of the opposite sex described in short verbal profiles. These profiles differed with respect to whether the described person made music or not. Additionally, the musicians’ profiles varied with regard to whether the described person played music or sang in public or in private only. Results show that musicians’ profiles were not generally rated as more attractive than non-musicians’, but attractiveness did vary according to setting: private musicians were rated as most attractive, followed by non-musicians and public musicians. Furthermore, results indicate that participants who played a musical instrument or sang themselves gave higher ratings to profiles of musicians. But for participants who do not make music themselves, higher attractiveness ratings for musicians playing instruments or sing in private settings were found. These results indicate that the impression of sharing a common interest (making music) and furthermore making music in private instrumental settings seems to make people attractive to other people. No additional support for the sexual selection hypotheses for the evolution of music was provided by the current results. The musical status of the rater affected his or her judgements, with musicians rating other people as more attractive if they share the common interest in making music. Not the display of being a musician seems to be critical for attractiveness ratings but the perceived or imagined similarity by the rater created by information on musicality, fostering the theoretical significance of the communication aspect of music.

## Introduction

Music has accompanied humans since the early beginnings of mankind. A flute made from the wing bone of a goose vulture, which was found at the Swabian Alb in 2009 and is estimated to be about 40,000 years old (Adler, 2009), is considered to be the oldest proof of human music-making. Cave paintings of similar age, depicting dancers and percussion instruments offer additional proof of the musical activities of our ancestors (Miller, 2000).

In today’s world, music is so omnipresent that its purpose in everyday life is not questioned. However, little is known about the origins and functions of music (Mosing et al., 2015; Killin, 2018). Why did our earliest ancestors invest their precious resources into musical activities? One possible answer is that music serves an evolutionary purpose by increasing a person’s reproductive success.

### The Evolution of Music

Evolution theory suggests that behaviors that lead to long-term reproductive advantages are maintained throughout the generations, while those with low benefit disappear, allowing for the maximization of relative reproductive success against the backdrop of limited resources (Darwin, 1871; Euler, 2014).

For a behavior to be considered a complex biological adaptation, it must meet certain criteria (Symons, 1990). First, it has to be a cross-cultural phenomenon, with historical traces to early human development. Furthermore, it must follow the same, standardized developmental process from one individual to the other. Additionally, every individual must potentially be able to learn the behavior and there must be evidence of genetic predisposition and the presence of a specific, responsible brain region (Miller, 2000; Schmitt and Pilcher, 2004).

Music behaviors meet these general criteria. For one, music is undoubtedly a cross cultural phenomenon. It serves as a means of emotional expression in all parts of the world and across all cultures (Dissanayake, 2006; Altenmüller and Bernatzky, 2015; Mehr et al., 2019). Even before prehistoric musical instruments were carved out of animal bone, the ultimate musical instrument existed: human voice (cf. Ewens, 1995; Foley, 2012). With its wide array of sounds and pitches – from deep humming to high whistles, the human voice is capable of communicating emotions and so lends itself to the creation of infinite melodies and songs. Further, studies have shown musical abilities such as motor music skills (Gilbert, 1980) and temporal accuracy (Smoll, 1974) to improve with chronological age, suggesting a standardized developmental process.

Further, almost any person can learn a melody or sing along the tune of their favorite commercial. With regard to the fifth criterion, hereditability, Trehub (2001) demonstrated that children paid more attention to their mother’s singing than to her speaking voice and recent finding have provided some evidence for genetic processes in music behaviors (Mehr et al., 2017). Finally, Peretz and Zatorre (2005) found links between musical activity and the activation of certain brain regions. They demonstrated that pitch-based (melodic) aspects of music predominantly involve the right auditory cortex, whereas extraction of time-based mechanisms (rhythms) recruits more widespread and bilateral neural networks. But there is no “music center” in the brain, rather there are always many brain regions involved in the complex processing of music. The study by Peretz and Zatorre (2005) is an example of the evidence that there is a neurophysiological basis for the perception of Music.

Music thus fulfills all prerequisites for being regarded as the result of complex biological adaptation (Darwin, 1871; Miller, 2000; Molino, 2000).

### Sexual Selection

In the archaic world of our ancestors, music-making came with considerable cost as it could attract predators or hostile clans, prevent young and weak group members from sleeping, and also required a lot of time for practice that could have been used, instead, for food gathering or for the fulfillment of other primary needs. From an evolutionary perspective, this excessive cost suggests that music-making must have carried some kind of advantage. If music is, indeed, the result of biological adaptation, two possible principles of evolution must be considered. While some postulate that music is the result of natural selection (Sloboda, 1985; Trehub, 2003), e.g., by means of increased survival due to better child bonding (Dissanayake, 2008; Fancourt and Perkins, 2018) or health (Kreutz et al., 2012; MacDonald et al., 2012), others argue in favor of music as a means of increasing reproductive success via sexual selection (Darwin, 1871; Miller, 2000).

Aside from the ability to create music, humans possess the channels necessary to perceive music made by others. Music can even elicit strong emotional reactions in the listener (Altenmüller and Bernatzky, 2015; Sakka and Juslin, 2018). Making music thus may serve a purpose by being addressed to a human recipient and thereby fostering emotional connections. Music as a means of forging and strengthening human bonds is in line with both the theory of natural selection and sexual selection. The latter, which views music as courting behavior, is further supported by the observation that making music is often closely linked to the motivation of constant improvement of one’s abilities. Similar to a male bird’s inexhaustible repetition of his song, musicians often find motivation in becoming “the best”, which is a powerful mechanism of sexual selection (Miller, 2001). This study aims at contributing further evidence of music as a means of increasing reproductive success.

Unlike the sexual selection of birds, human musicality exists in both sexes, in line with the observation that both males and females take part in human mating choices (Darwin, 1871). However, men and women pursue different strategies when choosing a partner (Buss, 1989). From an evolutionary point of view, resources and maturity are important partner characteristics for women in long-term relationships (Li and Kenrick, 2006). Men, on the other hand, place more emphasis on aspects such as fertility, youthfulness and health in long-term partners (Buss and Schmitt, 1993). The reproductive benefit of music-making should therefore be investigated separately for both sexes.

Only few studies exist which examine the effect of music-making on partner choice. Beneficial effects of music on sexual selection were found by Tifferet et al. (2012), who used Facebook profile pictures in their research, depicting a person with or without a guitar making friend requests. Profiles with guitars received almost three times as many positive responses as profiles without one. Further evidence of music as an attractive feature was found by Guéguen et al. (2014). In this study, a physically attractive actor standing in a pedestrian zone carrying either a guitar case, a sports bag or no bag at all, asked 300 women for their number. When carrying the guitar case, the man received twice as many phone numbers as when he carried no bag and three times as many as with the sports bag.

The present study addresses the reproductive advantage of music-making by comparing how attractive musicians and non-musicians are to members of the opposite sex. Attractiveness is measured via an online survey in which participants were presented with potential partners. Going beyond previous studies, we examine possible differences in attractiveness ratings for musicians who play a musical instrument versus musicians who sing, and for musicians who play professionally and in public versus musicians who only play in private settings.

When dealing with attractiveness assessments, it is important to consider the influence of common interests. As previous results show, recognized similarity can be a factor of attractiveness (Gebauer et al., 2012). Therefore, the present study also explores how the musical activity of the rater affects the perceived attractiveness of the musician.

Finally, the present study also addresses the potential influence of musical skill.

### Hypotheses

Based on the previous findings we expected attractiveness ratings to be higher for musicians’ profiles than for non-musicians’ profiles.

Due to a general preference for people who share the same interest in music, musicians were expected to receive higher attractiveness ratings by raters who played a musical instrument or sang themselves than by those who did not.

If music-making is, indeed, a form of courtship behavior originally intended to attract potential partners, individuals who are better at carrying out this ritual will be perceived as more attractive than those who are less skilled. Assuming that performing music in front of an audience requires a high level of skill, musicians who play in public are expected to be perceived as better and thus more attractive than those who only play or sing in private settings.

Finally, since it has been shown that musical aptitude is equally distributed across the sexes (Pollatou, 2005), no specific expectations existed with regard to possible gender effects, whether for attractiveness ratings or for raters’ gender.

## Materials and Methods

### Participants

In order to qualify for participation, participants had to be heterosexual, identify as either male or female and had to be between 16 and 40 years of age. A total of 169 participants meeting the criteria took part in this web-based survey. After the exclusion of 32 participants due to incomplete data, the final sample consisted of 137 individuals (95 female). Their age ranged from 16 to 39 years (*M* = 23.85; *SD* = 4.45). Fifty-eight stated that they were single, 65 were in a committed relationship, eight were married, five were in a casual affair and one person lived in an open relationship. About 42% (*n* = 58) reported to play a musical instrument or to sing.

Participants were recruited via distribution of the survey web link in social media. Participation was voluntary and no compensation was offered for the participation in this study. People interested in participating received information about the study’s purpose and procedure on the first page of the survey. All data were handled anonymously. Participants were free to terminate participation at any time. By continuing the survey, they gave informed consent to voluntary participation. No ethic review was required per institution guidelines and national regulations.

### Task and Material

Participants were presented with the descriptions of twelve fictive persons of the opposite sex via an online survey. The wording was chosen so as to resemble that used on dating websites (see Supplementary Material). The participants’ task was to rate the attractiveness of the described person. The descriptions contained the name, age, living situation, occupation and a few hobbies for each person.

Each description was designed in four versions with variations on two dimensions: (1) sex of the described person, by using a female or male first name, and (2) the person’s musical activity: half of the profiles described subjects who make music, half described ones who do not. One third of the musical profiles described people who only make music in the privacy of their own home, another third described people who play music in public but are not professional musicians, and the last third described professional musicians. Among the musically active profiles, half were described as playing an instrument, the other half was described as individuals who sing.

The following paragraph shows an example of a description in all four variants, male and female, with and without the musical attribute. In the non-musical condition, the text in brackets […] was left out. Thus, differences in attractiveness rating can be attributed solely to the added or omitted music information.

*Hiya, my name is Lea/Eric Dresdner, I am 21 years old and love nature more than anything in the world. I really enjoy short getaways by bike or on foot. I love picnics with friends and have a huge weakness for chocolate. At the moment I am studying nutritional science in Marburg, where I’m renting a small apartment. Most weekends I drive home to see my family and friends. [The choir that I sing in is also here in Frankfurt. We practice regularly and have performances now and* then. Singing is awesome.]

Participants were requested to read each profile thoroughly and state their interest in the following four activities: (1) getting to know the person, (2) going on a date with this person, (3) having sexual contact with the person, and (4) being in a relationship with the person. Participants marked their answers on a 6-point-scale ranging from 1 (*no interest at all*) to 6 (*very strong interest*). Further, participants had to rate whether the profile described an attractive person on a 6-point-scale ranging from 1 (*strongly disagree*) to 6 (*strongly agree*). In order to avoid the focus of the study on attractiveness becoming too salient, four additional masking traits (sympathetic, loyal, benevolent, intelligent) were to be rated.

### Procedures and Design

At the beginning of the survey, participants answered questions regarding demographic variables such as age, profession, sex, relationship status and sexual orientation. Items on age and profession were open format while items on sex, relationship status and preferred sex when choosing a partner used multiple-choice formats.

Afterward, participants were provided with a written instruction regarding the task. They were asked to read the twelve presented profiles thoroughly, answer the questions and assess the described person on the provided categories.

**FIGURE 1:**
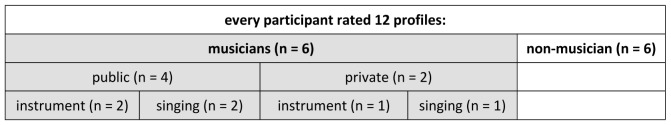
Nesting of profiles rated by participants.

Each participant was presented with twelve profiles describing persons of the opposite sex in randomized order. The order of profile presentations was defined by a computer random algorithm for each participant. Six of the twelve profiles for each participant described a musician and six did not mention music, leading to a within subject design. The musician profiles further included the two nested factors of instrument versus singing, and of public and private music-making (see Figure 1 and Supplementary Material for the design of profile characteristics).

After completion of the profiles-rating-task, participants were asked additional questions regarding their attitude to music (“Music is important in my daily routine”; “I would describe myself as a musician”; “Making music is a very important part of my life”; “It is very important to me that my partner also makes music”), partnership (“Financial resources matter in a partnership”; “In a relationship, the social status of my partner is important to me”; “I don’t care what other people think about my partner”; “It is important to me that other people also perceive my partner as attractive”; “Common hobbies are an important prerequisite for a good partnership”) and whether they regularly made music themselves.

The entire session lasted for about 15–20 min.

### Data Handling and Analyses

To determine the profiles’ attractiveness ratings, the ratings to the four questions regarding interest in the described person, and the task requiring the explicit rating of attractiveness for each fictitious person were added, forming one “profile attractiveness score”. Analyses yielded sufficient to excellent consistencies for each of the 48 profiles’ attractiveness ratings, with Cronbach’s α ranging from 0.74 to 0.94.

Group differences in ratings of attractiveness were analyzed using repeated measures Analyses of Variance (ANOVA). No differences were found between profiles describing musicians performing professionally and musicians performing publicly but as a hobby. Therefore, data for music performers were aggregated in subsequent analyses.

In addition, correlation analyses between the profiles’ attractiveness ratings and further items related to participants’ attitude to music and relationship status were conducted. For those subjects who make music themselves, musical affinity was also taken into account. A musical affinity scale was created by combining the following two items into one scale: “I would describe myself as a musician” and “Making music is a very important part of my life” (α = 0.89).

All analyses were conducted using IBM SPSS version 23 software package.

## Results

An ANOVA for repeated measures with participants’ musical status (making music themselves vs. not making music) and participants’ gender (male vs. female) as between-subject factors and musician- vs. non-musician profiles as a within-subject factor indicated that attractiveness ratings for musicians’ and non-musicians’ profiles did not differ, *F*(1,133) = 0.66; *p* = 0.42; η_p_^2^ = 0.005. Thus, participants viewed musicians and non-musicians as similarly attractive, regardless of the raters’ own musical status and gender.

**FIGURE 2:**
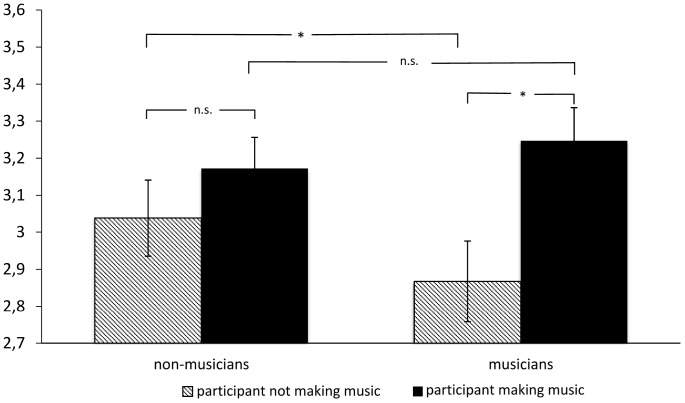
Attractiveness ratings for profiles of non-musicians and musicians by participants (Prt) making music and not making music themselves (error flags indicate standard errors of means; n.s., not significant; ^∗^*p* < 0.05).

However, participants who reported making music themselves rated the presented profiles as more attractive on the whole (*M* = 3.21; *SD* = 0.73) when compared to participants who did not play a musical instrument or sang, *M* = 2.95; *SD* = 0.69; *F*(1,133) = 4.30, *p* = 0.04; η_p_^2^ = 0.031. This main effect was further qualified by the group of musical profiles as indicated by a group-by-profiles-interaction [*F*(1,133) = 4.35; *p* = 0.039; η_p_^2^ = 0.032]. It was only the profiles describing musicians that were rated significantly more attractive by participants who made music themselves than by participants who did not play a musical instrument or sing (see Figure 2, right side), *post hoc* Bonferoni-test, *p* = 0.019. When rating non-musicians’ profiles, however, ratings by participants making music themselves did not differ significantly from those from participants who did not make music (Figure 2, left side; *p* = 0.362). Further, participants who did not make music themselves rated non-musicians’ profiles as more attractive than musician-profiles (Figure 2, gray columns, *p* = 0.030). Musically active participants, however, did not differentiate between profiles from musicians and non-musicians (Figure 2, black columns, *p* = 0.665).

Thus, both the musicians and the non-musicians described in the profiles benefited from the overall higher ratings provided by musically active participants. Both groups of raters tended to rate those profiles more highly, which shared their musical affinity, with this tendency being more pronounced in the group of non-musician raters.

The higher overall ratings by musically active participants are thus due to the group of music making participants who rated fellow musicians particularly highly and non-musicians particularly low.

No further main or interaction effect was found in this analysis, all *F*(1,133) < 0.82; *p* > 0.37; η_p_^2^ ≤ 0.006.

**FIGURE 3:**
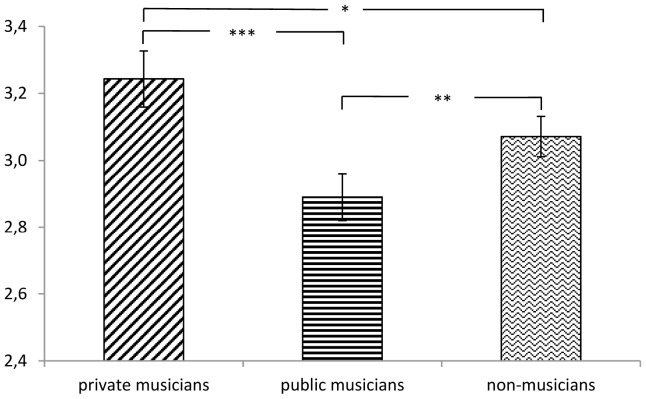
Means and standard errors for attractiveness ratings of profiles for private, public, and non-musicians. ^∗^*p* < 0.05, ^∗∗^*p* < 0.01, ^∗∗∗^*p* < 0.001.

Another ANOVA for repeated measures comparing three groups of profiles (private musicians, public musicians, and non-musicians) and using gender and participants’ musical status as between subject factors revealed a significant main effect, *F*(2,132) = 11.80; *p* < 0.001; η_p_^2^ = 0.152. The profiles of private musicians (*M* = 3.24, *SD* = 0.98) were rated as more attractive than profiles of non-musicians [*M* = 3.07, *SD* = 0.70; *t*(136) = 2.39, *p* = 0.018], and the latter were rated significantly higher than profiles of public musicians, *M* = 2.89, *SD* = 0.82; *t*(136) = 2.94, *p* = 0.004 (see Figure 3).

The previously reported effect for participants’ musical status was replicated in this analysis, with music-making participants giving higher average attractiveness ratings than participants not making music themselves, *F*(1,133) = 4.39; *p* = 0.04; η_p_^2^ = 0.032. No gender effect was found, *F*(1,133) = 1,59; *p* = 0.21. Thus, taking the music setting into account, we found musicians to be rated as more attractive in private settings only.

**FIGURE 4:**
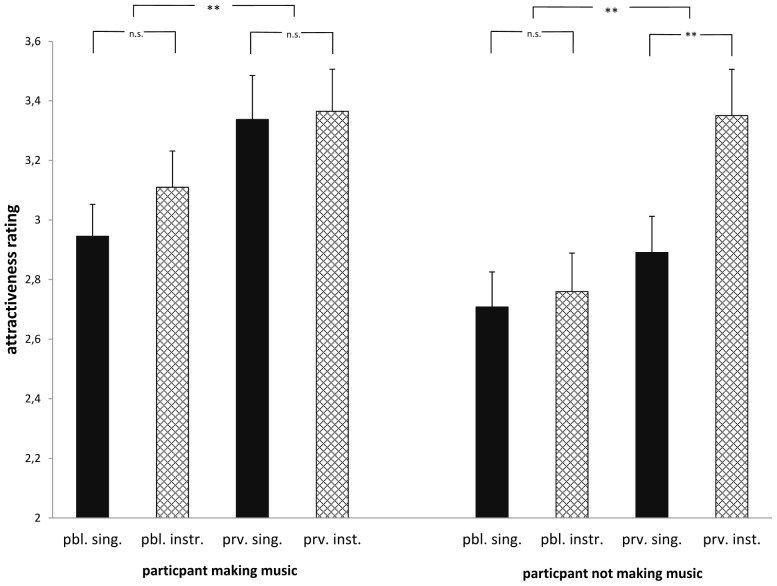
Attractiveness ratings from participants making music themselves or not making music themselves for profiles of musicians who sing or play an instrument in public or private settings (n.s., not significant; ^∗∗^*p* < 0.01).

A final ANOVA using music activity (playing an instrument vs. singing) and setting (private vs public performance) as within-subject factors and participants’ gender and participants’ musical status (making music themselves vs. not making music themselves) as between-subject factors replicated the previously found advantage of private musicians compared to public musicians, *F*(1,133) = 23.12; *p* < 0.001; η_p_^2^ = 0.148. This effect was further qualified by a significant three-way interaction between the variables publicity, musical activity, and participants’ musical status, *F*(1,133) = 4.56; *p* = 0.035; η_p_^2^ = 0.033. As can be seen from Figure 4, participants who do not make music themselves rated musicians who *play an instrument* in private settings as more attractive than musicians who *sing* in private settings, *t*(63) = 3.06; *p* = 0.003. However, when participants make music themselves, ratings for profiles of people who play an instrument vs sing in private settings did not differ, *t*(63) = 0.17; *p* = 0.87.

For the group of participants not making music themselves, the differences in ratings for private vs. public musicians is due to high ratings in private instrumental settings (see Figure 4). Further, as can be seen in Figure 4, ratings for musicians playing instruments in private did not differ depending on whether participants make music themselves or not [*F*(1,135) = 0.005; *p* = 0.945] while the ratings for musicians singing in private differed between both groups of participants, *F*(1,135) = 5.28; *p* = 0.023; η_p_^2^ = 0.038.

A trend for a gender-by-profile interaction [*F*(1,133) = 3.89; *p* = 0.051; η_p_^2^ = 0.03] suggests that the effect of a greater preference of private settings tended to be greater in men (*M* = 3.42 vs. *M* = 2.89) than in women (*M* = 3.17 vs. *M* = 2.89). No gender main effect was found in this analysis, *F*(1,133) = 1.48; *p* = 0.23.

A correlation analysis showed that participants’ musical affinity correlated moderately positively with the attractiveness ratings of the profiles, *r* = 0.38, *p* < 0.001. Thus, the more a participant conceptualized his or herself as a musician the more positive they rated the profiles of any person described as making music.

All ANOVAs described above were also conducted with status of relationship, participants’ sex, age, and subjective ratings of financial security, importance of partner’s social status, independence of other judgements on partner as covariates. None of these analyses produced any significant effect for the covariates (all *F* < 2.23; all *p* > 0.106; all η_p_^2^ < 0.020.

## Discussion

The present study examined a possible reproductive advantage of musicians based on psycho-evolutionary theories. In general, results failed to support the hypothesized higher attractiveness ratings for musicians compared to non-musicians. However, after taking the setting into account, we found musicians who played an instrument or sang in a non-public setting were rated as more attractive than non-musicians while public musicians were rated as least attractive. Thus, the failure to find a general advantage for musicians is due to the low attractiveness of public musicians, especially in the eyes of male raters.

From an evolutionary perspective, music-making has been interpreted as courting behavior (Darwin, 1871; Miller, 2000) and one might suspect public courting behavior to be less appreciated than private courting. A listener might feel less courted by public music performances while the exclusiveness of a private music performance may more readily be interpreted by the listener as courting behavior.

Further, public music-making is often associated with distress, the pursuit of perfectionism and the fear of failure (Jäncke, 2011; Gembris et al., 2018). As a result, the high burden on health leaves less resources for partner and family, which might make the person appear less attractive from an evolutionary perspective. Unfortunately, we did not ask participants to rate the musical abilities they ascribe to the person characterized in the profiles. Therefore, we can only speculate at this point about what participants might have associated with persons making music in public settings. Future research should therefore additionally assess assigned musical ability in order to rule out that reported effects are confounded by such a similarity effect demonstrated in our study.

Taking participants’ own musical status into account, results revealed both groups of raters - those who were musically active and those who were not - to provide significantly higher ratings to profiles which resembled their own status regarding musical activity. Thus, participants who did not make music themselves rated non-musicians profiles as more attractive than musician-profiles. And while the effect did not reach statistical significance, participants who made music did show a tendency of rating fellow musicians’ profiles highest. This is supported by correlation analyses which revealed that those musicians whose music affinity was particularly high, meaning that music plays a particularly important role in their lives, were more likely to rate the profiles of fellow musicians as more attractive. This findings support the assumption that the shared interest in music (or lack thereof) has a positive effect on the perceived attractiveness of a potential partner. Buss (1984) drew up the similarity-attracts hypothesis, which assumes that people prefer mates who are similar to themselves. The assumption that common interests generally play a role in choice of mate has since been supported by many studies (e.g., Watson et al., 2004; Zietsch et al., 2011; Gebauer et al., 2012). Therefore, future studies investigating attractiveness of musicians should always also assess participants’ musical status. In future research, rater status should also include whether the rater plays music in public or private settings and whether the rater plays a musical instrument or sings.

Interestingly, within the profiles of private instrumental musicians, the effect of common interests is not sufficient. Whether participants made music themselves or not did not influence their attractiveness ratings for private instrumental musician profiles. Thus, common interests do not seem to be the only factor in explaining perceived attractiveness.

An additional finding was that participants who made music themselves rated all profiles as more attractive when compared to participants who do not make music. These rating differences between musicians and non-musicians were more pronounced when participants rated profiles of musicians.

Previous findings supporting the reproductive advantage of musical activity via Facebook profiles (Tifferet et al., 2012) or carrying a guitar case (Guéguen et al., 2014) are, thus, not supported by our data. Our study used verbal, written profiles of fictitious persons which differed from the respective control condition in only a few words. Thus, it might be that our variations were less salient to participants and the effect of our profiles depended more on the internal images created in our participants by those profiles.

As stated above, Miller (2000) presumes that – similar to the bird’s song – human music-making may not be a conscious attempt at gaining a reproductional advantage. According to Wedekind et al. (1995) mate choice is predominantly an unconscious selection process. In contrast, the present study asked the subjects to explicitly deal with the attractiveness of a potential partner. Subjects were asked to read carefully, and their attention was explicitly drawn to the feature of attractiveness. This cognitive activity might have interfered with the subconscious process of choosing a partner.

Further, music has often been considered similar to language and its capacities as a means of communication have been emphasized (e.g., Marler, 2000; Cross, 2009). Thus, beyond just seeing similarities between themselves and the profiled person, participants might have recognized a common “language” in music which facilitates communication. Communication in a private setting can be assumed to be more effective and more intimate. Therefore, this study does not provide evidence for the bird’s colored feathers metaphor of music in sexual selection but it provides results which are in accordance with a communication framework for the evolution of music (Cross and Morley, 2008; Schulkin and Raglan, 2014).

A challenge of the present investigation was to capture the effect of a profile’s musical attribute independently of other influences. An attempt was made to prevent the question of musical activity from being too noticeable for the participants, while still being sufficiently salient to influence attractiveness ratings. To achieve this goal, musical attributes were presented in different variations of private, public or professional musicians. In order to keep the demands for our participants reasonable, we decided to limit the total number of profiles rated by each volunteer to twelve. However, this concept led to small subgroups for each variation of the profiles regarding the distinction between non-musician, private-musician and public-musician. Nevertheless, the results confirm that this study had sufficient power.

To achieve high internal validity, various measures have been taken to reduce confounding influences. First, two identical profiles were used which differed only with regard to the presence of a musical attribute. All other attributes of the profiles were kept as average and unpolarizing as possible with regard to attractiveness. Second, the chosen within-subject design was supposed to exclude personal confounding variables of participants. Effects of possible remaining confounders were to be equalized by randomizing the allocation of profiles to both experimental groups. Nevertheless, we cannot prove that other possible attributes to the profiles might have been in effect too and therefore, for future research, we recommend examining additional variables like the social status or earning potential attributed to the profiles. This could offer some additional approaches of explanation, why private musicians are perceived as more attractive and all musicians would be less discriminating in partner selection.

In sum, this study provides evidence that an individual’s musical status might influence the beholder’s judgment albeit not always in the beneficial way, being a musician may even prove to be a disadvantage. The present data revealed an additional way in which music may influence the dating process: the musical status of the rater affects his or her judgements, with musicians rating other people as more attractive on the whole, particularly if music is a common interest.

Not the display of being a musician seems to be critical for attractiveness ratings but the perceived or imagined similarity by the rater created by information on musicality. This outcome emphasizes the significance of communication in the evolution of music.

## Data Availability Statement

The datasets generated for this study are available on request to the corresponding author.

## Ethics Statement

Ethical review and approval was not required for the study on human participants in accordance with the local legislation and institutional requirements. The participants provided their written informed consent to participate in this study.

## Author Contributions

All authors listed have made a substantial, direct and intellectual contribution to the work, and approved it for publication.

## Conflict of Interest

The authors declare that the research was conducted in the absence of any commercial or financial relationships that could be construed as a potential conflict of interest.
