# Supplementary material for: Attractiveness Ratings for Musicians and Non-musicians: An Evolutionary-Psychology Perspective
Source: Front Psychol. 2019 Nov 29;10:2627. doi: 10.3389/fpsyg.2019.02627 (PMC6895061; doi:10.3389/fpsyg.2019.02627)
Supplement: Supplementary file 1 [file Data_Sheet_1.PDF]

OVERVIEW OF ALL PROFILES, THE ASSOCIATED VARIABLES AND THE PROFILES' RELIABILITIES

First music, then no music				First no music, then music			
**Female Sample**							
Gr.1 – male profiles n = 46			reliability	Gr.2 – male profiles n = 49			reliability
v_ 6; v_ 7	1_A_M_P_I	Lukas Schneider	0,925	v_30; v_31	2_a_m	Lukas Schneider	0,923
v_ 8; v_ 9	1_B_M_K_I	Ralph Eberhardt	0,925	v_32; v_33	2_b_m	Ralph Eberhardt	0,824
v_10; v_11	1_C_M_B_I	Christian Löwe	0,943	v_34; v_35	2_c_m	Christian Löwe	0,921
v_12; v_13	1_D_M_P_S	Matthias König	0,91	v_36; v_37	2_d_m	Matthias König	0,919
v_14; v_15	1_E_M_K_S	Vincent Weber	0,934	v_38; v_39	2_e_m	Vincent Weber	0,911
v_16; v_17	1_F_M_B_S	Jonas Stempe	0,916	v_40; v_41	2_f_m	Jonas Stempe	0,909
v_18; v_19	1_g_m	Manuel Kaufner	0,938	v_42; v_43	2_G_M_P_I	Manuel Kaufner	0,938
v_20; v_21	1_h_m	Karl Lose	0,911	v_44; v_45	2_H_M_K_I	Karl Lose	0,916
v_22; v_23	1_i_m	Alexander Kaiser	0,934	v_46; v_47	2_I_M_B_I	Alexander Kaiser	0,944
v_24; v_25	1_j_m	Valentin Mayer	0,902	v_48; v_49	2_J_M_P_S	Valentin Mayer	0,874
v_26; v_27	1_k_m	Eric Dresdner	0,934	v_50; v_51	2_K_M_K_S	Eric Dresdner	0,922
v_28; v_29	1_l_m	Niklas Krause	0,89	v_52; v_53	2_L_M_B_S	Niklas Krause	0,901
**Male Sample**							
Gr.3 – female profiles = 21			reliability	Gr. 4 – female profiles n = 21			reliability
v_54; v_55	3_A_F_P_I	Lisa Schneider	0,9	v_ 78; v_ 79	4_a_f	Lisa Schneider	0,867
v_56; v_57	3_B_F_K_I	Brigitte Eberhardt	0,916	v_ 80; v_ 81	4_b_f	Brigitte Eberhardt	0,797
v_58; v_59	3_C_F_B_I	Bianca Löwe	0,892	v_ 82; v_ 83	4_c_f	Bianca Löwe	0,875
v_60; v_61	3_D_F_P_S	Luisa König	0,924	v_ 84; v_ 85	4_d_f	Luisa König	0,859
v_62; v_63	3_E_F_K_S	Karina Weber	0,909	v_ 86; v_ 87	4_e_f	Karina Weber	0,928
v_64; v_65	3_F_F_B_S	Nicole Stempe	0,876	v_ 88; v_ 89	4_f_f	Nicole Stempe	0,841
v_66; v_67	3_g_f	Manuela Kaufner	0,929	v_ 90; v_ 91	4_G_F_P_I	Manuela Kaufner	0,888
v_68; v_69	3_h_f	Franziska Lose	0,922	v_ 92; v_ 93	4_H_F_K_I	Franziska Lose	0,938
v_70; v_71	3_i_f	Aileen Kaiser	0,855	v_ 94; v_ 95	4_I_F_B_I	Aileen Kaiser	0,872
v_72; v_73	3_j_f	Corinna Mayer	0,866	v_ 96; v_ 97	4_J_F_P_S	Corinna Mayer	0,884
v_74; v_75	3_k_f	Lea Dresdner	0,914	v_ 98; v_ 99	4_K_F_K_S	Lea Dresdner	0,741
v_76; v_77	3_l_f	Martina Krause	0,917	v_100; v_101	4_L_F_B_S	Martina Krause	0,895

PROFILE CODING:

Position 1: Number of the experimental group (1-4)
- *1+2*: Male Profiles for the female sample; *3+4*: Female profiles for the male sample

Position 2: Profile (A-L/a-l)
- *Capital letters:* Music; *Small letters:* No music

Position 3: Sex (M/m/W/w)
- *M/m = male profile; W/w = female profile*

If making music:

Position 4: Musical context (P/K/B)
- *P = Recreational music-making (private); K = Perfoms in concerts (public); B = Professional music-making* (public)

Position 5: Way of making music (I/S)
- *I* = Instrumental; *S* = Singing

P R O F I L E S

A. (P_I) Lukas /Lisa Schneider

- 27 years
- Profession: Retail dealer
- Living situation: one-bedroom apartment on the outskirts of Cologne
- Hobbies:
  - Soccer club on the weekend
  - Photography
  - *Playing the guitar*

1_A_M_P_I
Hi, my name is Lukas Schneider, I am 27 years old and have been living in a pretty little apartment in the Bornheim neighborhood of Frankfurt for 2 years. I work as a retail dealer in the textile industry. I enjoy photography and love to stay at home and jam on my guitar for hours on end. I am also an avid soccer player and like to play in tournaments with my team on the weekends.

2_a_m
Hi, my name is Lukas Schneider, I am 27 years old and have been living in a pretty little apartment in the Bornheim neighborhood of Frankfurt for 2 years. I work as a retail dealer in the textile industry. I enjoy photography. I am also an avid soccer player and like to play in tournaments with my team on the weekends.

3_A_F_P_I
Hi, my name is Lisa Schneider, I am 27 years old and have been living in a pretty little apartment in the Bornheim neighborhood of Frankfurt for 2 years. I work as a retail dealer in the textile industry. I enjoy photography and love to stay at home and jam on my guitar for hours on end. I am also an avid soccer player and like to play in tournaments with my team on the weekends.

4_a_f
Hi, my name is Lisa Schneider, I am 27 years old and have been living in a pretty little apartment in the Bornheim neighborhood of Frankfurt for 2 years. I work as a retail dealer in the textile industry. I enjoy photography. I am also an avid soccer player and like to play in tournaments with my team on the weekends.

B. (K_I) Ralph / Brigitte Eberhardt

- 22 years
- Profession: Hairdresser
- Living situation: Kronberg, small apartment
- Hobbies:
    - Sociable → Board games with friends
    - Cooking for friends
    - *Plays concerts with his band as a guitarist*

1_B_M_K_I
Cheers, my name is Ralph Eberhardt. I am 22 and work as a self-employed hairdresser in Kronberg. I have a small apartment there. I consider myself to be very sociable and enjoy playing board games in good company. I also love to cook for others. Playing the guitar is also a big hobby of mine. Now and then I play in concerts with my band.

2_b_m
Cheers, my name is Ralph Eberhardt. I am 22 and work as a self-employed hairdresser in Kronberg. I have a small apartment there. I consider myself to be very sociable and enjoy playing board games in good company. I also love to cook for others.

3_B_F_K_I
Cheers, my name is Brigitte Eberhardt. I am 22 and work as a self-employed hairdresser in Kronberg. I have a small apartment there. I consider myself to be very sociable and enjoy playing board games in good company. I also love to cook for others. Playing the guitar is also a big hobby of mine. Now and then I play in concerts with my band.

4_b_f
Cheers, my name is Brigitte Eberhardt. I am 22 and work as a self-employed hairdresser in Kronberg. I have a small apartment there. I consider myself to be very sociable and enjoy playing board games in good company. I also love to cook for others.

C. (B_I) Christian / Bianca Löwe

- 27 years
- Profession: *Concert pianist* / film producer → on the road a lot
- Living situation: Frankfurt Sachsenhausen
- Hobbies:
  - Gardening
  - Dog "Sasu"
  - Meeting friends at bars

1_C_M_B_I
Hi, I am Christian Löwe, 27, from Frankfurt. I live in a cozy apartment in Sachsenhausen. I am a concert pianist and play for various bands with whom I often go on tour. I also play in a band in my free time. We cover songs and often give concerts for small events. During tour breaks I love to garden in Frankfurt's city garden and spend my evenings going out to bars with friends. My dog, Sasu, is a fixture in my life and follows me everywhere.

2_c_m
Hi, I am Christian Löwe, 27, from Frankfurt. I live in a cozy apartment in Sachsenhausen. I work as a production assistant for different film production companies. I often travel for several weeks at a time for work. In between shoots, I love to garden in Frankfurt's city garden and spend my evenings going out to bars with friends. My dog, Sasu, is a fixture in my life and follows me everywhere.

3_C_F_B_I
Hi, I am Bianca Löwe, 27, from Frankfurt. I live in a cozy apartment in Sachsenhausen. I am a concert pianist and play for various bands with whom I often go on tour. I also play in a band in my free time. We cover songs and often give concerts for small events. In between shoots, I love to garden in Frankfurt's city garden and spend my evenings going out to bars with friends. My dog, Sasu, is a fixture in my life and follows me everywhere.

4_c_f
Hi, I am Bianca Löwe, 27, from Frankfurt. I live in a cozy apartment in Sachsenhausen. I work as a production assistant for different film production companies. I often travel for several weeks at a time for work. In between shoots, I love to garden in Frankfurt's city garden and spend my evenings going out to bars with friends. My dog, Sasu, is a fixture in my life and follows me everywhere.

D. (P_S) Matthias /Luisa König

- 24 years
- Profession: Journalist
- Living situation: On location in Frankfurt
- Hobbies:
  - Drawing
  - *Always singing in my free time*
  - Climbing, e.g. while on vacation, traveling, nature

1_D_M_P_S
Hi, my name is Matthias König. I am 24 and live in a suburb of Frankfurt. In Frankfurt I work as a freelance journalist for a newspaper. I really like to draw and am generally quite creative. Singing is my passion – I sing every free minute I get. Apart from that I spend my free time climbing. Even during my vacations I like to go to the mountains in order to go climbing there and enjoy nature.

2_d_m
Hi, my name is Matthias König. I am 24 and live in a suburb of Frankfurt. In Frankfurt I work as a freelance journalist for a newspaper. I really like to draw and am generally quite creative. Apart from that I spend my free time climbing. Even during my vacations I like to go to the mountains in order to go climbing there and enjoy nature.

3_D_F_P_S
Hi, my name is Luisa König. I am 24 and live in a suburb of Frankfurt. In Frankfurt I work as a freelance journalist for a newspaper. I really like to draw and am generally quite creative. Singing is my passion – I sing every free minute I get. Apart from that I spend my free time climbing. Even during my vacations I like to go to the mountains in order to go climbing there and enjoy nature.

4_d_f
Hi, my name is Luisa König. I am 24 and live in a suburb of Frankfurt. In Frankfurt I work as a freelance journalist for a newspaper. I really like to draw and am generally quite creative. Apart from that I spend my free time climbing. Even during my vacations I like to go to the mountains in order to go climbing there and enjoy nature.

E. (K_S) Vincent / Karina Weber

- 23 years
- Profession: studying to become a secondary school teacher
- Living situation: lives with parents in Bamberg
- Hobbies:
  - Traveling to foreign countries
  - *Singing in a choir (also giving concerts)*
  - Other: volunteer work for refugees

1_E_M_K_S
Hello, I am Vincent Weber. I am 23 years old and am currently studying to become a secondary school teacher. I still live with my parents near Frankfurt. I am a member of a gospel choir with which I have performances now and then. In my free time I also volunteer for refugee projects. During the semester break I try to travel as much as I can.

2_e_m
Hello, I am Vincent Weber. I am 23 years old and am currently studying to become a secondary school teacher. I still live with my parents near Frankfurt. In my free time I also volunteer for refugee projects. During the semester break I try to travel as much as I can.

3_E_F_K_S
Hello, I am Karina Weber. I am 23 years old and am currently studying to become a secondary school teacher. I still live with my parents near Frankfurt. I am a member of a gospel choir with which I have performances now and then. In my free time I also volunteer for refugee projects. During the semester break I try to travel as much as I can.

4_e_f
Hello, I am Karina Weber. I am 23 years old and am currently studying to become a secondary school teacher. I still live with my parents near Frankfurt. In my free time I also volunteer for refugee projects. During the semester break I try to travel as much as I can.

F. (B_S) Jonas /Nicole Stempe

- 21 years
- Profession: *Background singer*/Graffiti artist
- Living situation: Frankfurt city center
- Hobbies:
    - Repairing old cars
    - Mountain-biking
    - Basketball

1_F_M_B_S
Hi, I am Jonas Stempe and I am 21 years old. I have been singing for as long as I can remember and I started working as a professional background singer after graduating high school. Frankfurt is my home base. I have a small apartment here, right in the center of town. I ride my bike most places, and also love off-road cycling. I meet my boys to play basketball. Another hobby of mine is tinkering around with old cars at my buddy's workshop.

2_f_m
Hi, I am Jonas Stempe and I am 21 years old. I am a freelance artist and graffiti sprayer. Frankfurt is my home base. I have a small apartment here, right in the center of town. I ride my bike most places, and also love off-road cycling. I meet my boys to play basketball. Another hobby of mine is tinkering around with old cars at my buddy's workshop.

3_F_F_B_S
Hi, I am Nicole Stempe and I am 21 years old. I have been singing for as long as I can remember and I started working as a professional background singer after graduating high school. Frankfurt is my home base. I have a small apartment here, right in the center of town. I ride my bike most places, and also love off-road cycling. I meet my boys to play basketball. Another hobby of mine is tinkering around with old cars at my buddy's workshop.

4_f_f
Hi, I am Nicole Stempe and I am 21 years old. I am a freelance artist and graffiti sprayer. Frankfurt is my home base. I have a small apartment here, right in the center of town. I ride my bike most places, and also love off-road cycling. I meet my boys to play basketball. Another hobby of mine is tinkering around with old cars at my buddy's workshop.

G. (P_I) Manuel/Manuela Kaufner

- 32 years
- Profession: Architect
- Living situation: currently searching in pretty Vordertaunus
- Hobbies:
    - Cooking
    - *Playing the piano*

1_g_m
Hallo, my name is Manuel Kaufner and I am 32 years old. I am in the greater Frankfurt area a lot for work as an architect and like the beautiful Vordertaunus best. I would love to live there and am currently searching for a nice, small apartment there. After work I like to cook and try many new recipes.

2_G_M_P_I
Hallo, my name is Manuel Kaufner and I am 32 years old. I am in the greater Frankfurt area a lot for work as an architect and like the beautiful Vordertaunus best. I would love to live there and am currently searching for a nice, small apartment there. After work I like to cook and try many new recipes. I am also a passionate piano player because playing really helps me unwind.

3_g_f
Hallo, my name is Manuela Kaufner and I am 32 years old. I am in the greater Frankfurt area a lot for work as an architect and like the beautiful Vordertaunus best. I would love to live there and am currently searching for a nice, small apartment there. After work I like to cook and try many new recipes.

4_G_F_P_I
Hallo, my name is Manuela Kaufner and I am 32 years old. I am in the greater Frankfurt area a lot for work as an architect and like the beautiful Vordertaunus best. I would love to live there and am currently searching for a nice, small apartment there. After work I like to cook and try many new recipes. I am also a passionate piano player because playing really helps me unwind.

H. (K_I) Karl/Franziska Loser

- 25 years
- Profession: police officer
- Comes from Hamburg
- Hobbies:
  - *Saxophone in a big band – loves performances*
  - Geocaching – investigative skill

1_h_m
Moin moin, my name is Karl Loser. I am 25 years young, originally from beautiful Hamburg, and completed my training to become a policeman last year. Since I have always enjoyed geocaching, solving riddles, and collecting clues, I turned my hobby into my dream job.

2_H_M_K_I
Moin moin, my name is Karl Loser. I am 25 years young, originally from beautiful Hamburg, and completed my training to become a policeman last year. Since I have always enjoyed geocaching, solving riddles, and collecting clues, I turned my hobby into my dream job. Twice a week I play the saxophone in a big band. We have performances every couple of weeks, which I find great.

3_h_f
Moin moin, my name is Franziska Loser. I am 25 years young, originally from beautiful Hamburg, and completed my training to become a policewoman last year. Since I have always enjoyed geocaching, solving riddles, and collecting clues, I turned my hobby into my dream job.

4_H_F_K_I
Moin moin, my name is Franziska Loser. I am 25 years young, originally from beautiful Hamburg, and completed my training to become a policewoman last year. Since I have always enjoyed geocaching, solving riddles, and collecting clues, I turned my hobby into my dream job. Twice a week I play the saxophone in a big band. We have performances every couple of weeks, which I find great.

I. (B_I) Alexander/Aileen Kaiser

- 30 years
- Profession: *Lecturer for guitar studies*/graphic design
- Hobbies:
  - Traveling to new cities and countries
  - Biggest wish: trip around the world

1_i_m
Hello, I am Alexander Kaiser and am 30 years old. Even as a teen I was very interested in art and design. A few years ago I was lucky enough to make a career out of my passion as a lecturer for graphic design at Frankfurt University of Applied Sciences. When I am not busy being creative, I really like to head out and explore new cities and countries. My biggest wish is to really take the time and travel around the world some day.

2_I_M_B_I
Hello, I am Alexander Kaiser and am 30 years old. I started playing the electric guitar in my teens and was always very passionate about it. A few years ago I was lucky enough to make a career out of my hobby as a lecturer for guitar studies at Frankfurt University of Applied Sciences. In addition to teaching, I am often booked as an electric guitarist by bands for various performances. When I am not busy making music, I really like to head out and explore new cities and countries. My biggest wish is to really take the time and travel around the world some day.

3_i_f
Hello, I am Aileen Kaiser and am 30 years old. Even as a teen I was very interested in art and design. A few years ago I was lucky enough to make a career out of my passion as a lecturer for graphic design at Frankfurt University of Applied Sciences. When I am not busy being creative, I really like to head out and explore new cities and countries. My biggest wish is to really take the time and travel around the world some day.

4_I_F_B_I
Hello, I am Aileen Kaiser and am 30 years old. I started playing the electric guitar in my teens and was always very passionate about it. A few years ago I was lucky enough to make a career out of my hobby as a lecturer for guitar studies at Frankfurt University of Applied Sciences. In addition to teaching, I am often booked as an electric guitarist by bands for various performances. When I am not busy making music, I really like to head out and explore new cities and countries. My biggest wish is to really take the time and travel around the world some day.

J. (P_S) Valentin/Corinna Mayer
• 31 years
• Profession: Interpreter
• Hobbies:
- Sport: team handball, jogging, cycling
- *Singing*
- Volunteering: Animal shelter

1_j_m
Hi, my name is Valentin Mayer, I am 31 years old and am employed as an interpreter by a small corporation in Friedberg. In my free time I do tons of sports, I play handball on a team, like jogging and love taking long bike rides with my friends on the weekends. On the side, I volunteer at an animal shelter in town.

2_J_M_P_S
Hi, my name is Valentin Mayer, I am 31 years old and am employed as an interpreter by a small corporation in Friedberg. In my free time I do tons of sports, I play handball on a team, like jogging and love taking long bike rides with my friends on the weekends. Additionally, I am a passionate singer. Several friends have even told me to audition for DSDS, a singing competition television series. We'll see, I might actually try that one day. On the side, I volunteer at an animal shelter in town.

3_j_f
Hi, my name is Corinna Mayer, I am 31 years old and am employed as an interpreter by a small corporation in Friedberg. In my free time I do tons of sports, I play handball on a team, like jogging and love taking long bike rides with my friends on the weekends. On the side, I volunteer at an animal shelter in town.

4_J_F_P_S
Hi, my name is Corinna Mayer, I am 31 years old and am employed as an interpreter by a small corporation in Friedberg. In my free time I do tons of sports, I play handball on a team, like jogging and love taking long bike rides with my friends on the weekends. Additionally, I am a passionate singer. Several friends have even told me to audition for DSDS, a singing competition television series. We'll see, I might actually try that one day. On the side, I volunteer at an animal shelter in town.

K. (K_S) Eric/Lea Dresdner

- 21 years
- Profession: Nutritional science student
- Living situation: small apartment in Marburg, home at the weekends
- Hobbies:
    - Nature lover – taking small trips
    - Weakness for chocolate
    - *Singing in a choir*

1_k_m
Hiya, my name is Eric Dresdner, I am 21 years old and love nature more than anything in the world. I really enjoy short getaways by bike or on foot. I love picnics with friends and have a huge weakness for chocolate. At the moment I am studying nutritional science in Marburg, where I'm renting a small apartment. Most weekends I drive home to see my family and friends.

2_K_M_K_S
Hiya, my name is Eric Dresdner, I am 21 years old and love nature more than anything in the world. I really enjoy short getaways by bike or on foot. I love picnics with friends and have a huge weakness for chocolate. At the moment I am studying nutritional science in Marburg, where I'm renting a small apartment. Most weekends I drive home to see my family and friends. The choir that I sing in is also here in Frankfurt. We practice regularly and have performances now and then. Singing is awesome.

3_k_f
Hiya, my name is Lea Dresdner, I am 21 years old and love nature more than anything in the world. I really enjoy short getaways by bike or on foot. I love picnics with friends and have a huge weakness for chocolate. At the moment I am studying nutritional science in Marburg, where I'm renting a small apartment. Most weekends I drive home to see my family and friends.

4_K_F_K_S
Hiya, my name is Eric Dresdner, I am 21 years old and love nature more than anything in the world. I really enjoy short getaways by bike or on foot. I love picnics with friends and have a huge weakness for chocolate. At the moment I am studying nutritional science in Marburg, where I'm renting a small apartment. Most weekends I drive home to see my family and friends. The choir that I sing in is also here in Frankfurt. We practice regularly and have performances now and then. Singing is awesome.

L. (B_S) Niklas/Martina Krause

- 24 years
- Profession: *Performer in a musical* / stage actor
- Living situation: One-bedroom apartment in Frankfurt Höchst
- Hobbies:
  - Reading
  - Watching tv
  - Prefer quiet evenings on my couch

1_l_m
Hello, Niklas Krause's the name. I am 24 years old and live in a small one-bedroom apartment in Frankfurt Höchst. Just a short while ago I graduated from acting school and have since been working at a theater in Frankfurt. That's a ton of fun. It's just a great feeling to witness the audience's enthusiasm. After a long day of work I like to relax on my sofa while reading or watching tv, and will rarely object to staying in and enjoying a quiet evening.

2_L_M_B_S
Hello, Niklas Krause's the name. I am 24 years old and live in a small one-bedroom apartment in Frankfurt Höchst. Just a short while ago I completed training to become a musical performer and have since been working as a member of a free musical ensemble in Frankfurt. Singing on stage is a ton of fun. It's just a great feeling to witness the audience's enthusiasm. After a long day of work I like to relax on my sofa while reading or watching tv, and will rarely object to staying in and enjoying a quiet evening.

3_l_f
Hello, Martina Krause's the name. I am 24 years old and live in a small one-bedroom apartment in Frankfurt Höchst. Just a short while ago I graduated from acting school and have since been working at a theater in Frankfurt. That's a ton of fun. It's just a great feeling to witness the audience's enthusiasm. After a long day of work I like to relax on my sofa while reading or watching tv, and will rarely object to staying in and enjoying a quiet evening.

4_L_F_B_S
Hello, Martina Krause's the name. I am 24 years old and live in a small one-bedroom apartment in Frankfurt Höchst. Just a short while ago I completed training to become a musical performer and have since been working as a member of a free musical ensemble in Frankfurt. Singing on stage is a ton of fun. It's just a great feeling to witness the audience's enthusiasm. After a long day of work I like to relax on my sofa while reading or watching tv, and will rarely object to staying in and enjoying a quiet evening.
